# *Helicobacter pylori* Exposure in Nausea and Vomiting of Pregnancy Increases Risk of Preterm Delivery

**DOI:** 10.1155/2023/6612268

**Published:** 2023-09-28

**Authors:** Amr H. Masaadeh, Patrick C. Mathias, Bradley A. Ford, Dustin E. Bosch

**Affiliations:** ^1^Department of Pathology, Roy J. and Lucille A. Carver College of Medicine, University of Iowa, Iowa City, IA, USA; ^2^Department of Laboratory Medicine and Pathology, University of Washington, Seattle, WA, USA

## Abstract

**Background:**

Hyperemesis gravidarum (HG), a severe form of nausea and vomiting in pregnancy (NVP), is a leading indication for hospitalization in the first trimester. NVP and HG are associated with *Helicobacter pylori* (HP) infection in non-United States cohorts. How HP exposure and NVP interact to affect metabolic disturbance and pregnancy outcomes is not known.

**Materials and Methods:**

We designed a retrospective cohort study relating HP and NVP to serum electrolyte laboratory results, preterm delivery, and infant birth weight. Single academic institution discovery and independent multi-institutional validation cohorts included pregnant subjects with an HP test result. Associations of HP, NVP, and pregnancy outcomes were assessed with odds ratio calculations, Student's *t*-tests, and multivariate logistic regression.

**Results:**

Among subjects with positive HP test results, the prevalence of hyperemesis gravidarum (HG) was 0.025 (66 of 2671) and NVP was 0.27 (710 of 2671). Subjects with negative HP had prevalence of HG 0.015 (165 of 10,960) and NVP 0.22 (2392 of 10,960). History of HP exposure increased risk of NVP, including HG (odds ratio 1.3, 95% CI 1.1-1.4). Patients with HP exposure had lower serum potassium (mean difference 0.1 mEq/L) and bicarbonate (mean difference 0.3 mEq/L) during pregnancy than HP-negative patients (*p* < 0.01). Serum potassium was lowest in subjects with both NVP and HP exposure (mean 3.5 mEq/L [3.4-3.6], *p* < 0.0001). HP exposure alone carried increased risk for preterm delivery (OR 1.3 [1.1-1.4]). NVP alone increased risk of preterm delivery (OR 2.8 [2.5-3.1]) including second trimester delivery (OR 2.2 [1.7-2.8]). In multivariate analysis, HP exposure in the setting of NVP further increased risk of preterm delivery (adjusted OR 1.4 [1.0-1.9], *p* = 0.03).

**Conclusions:**

*H. pylori* exposure and diagnosis of NVP are individually associated with metabolic disturbances and adverse pregnancy outcomes such as preterm labor and delivery, and their combination further increases risk in US populations.

## 1. Introduction

Nausea and vomiting of pregnancy affects approximately 70% of patients in pregnancy [1]. The most severe form of this disease spectrum, hyperemesis gravidarum (HG), occurs in roughly 1% of pregnancies and is a leading indication for hospitalization in the first trimester [2, 3]. Universally agreed-upon diagnostic criteria for HG are lacking, but many of the definitions include severe nausea and vomiting associated with weight loss, dehydration, and/or electrolyte imbalances [4]. In severe or untreated cases, HG can lead to nutritional deficiencies such as vitamin B1 deficiency, electrolyte imbalances including hypokalemia, and effects on maternal psychiatric health [1–3]. The relationship of HG to fetal and neonatal outcomes has been controversial, in part because of observational study designs, nonuniform criteria for diagnosis of HG, and varying approaches to management [4, 5]. One very large English observational cohort study identified modest effect-size associations (odds ratios [OR] 1-2) of HG with maternal anemia, preeclampsia, eclampsia, venous thromboembolism, induction of labor, preterm delivery, and low birthweight [2].

A global systematic review in 2015 estimated that 4.4 billion individuals were infected with *Helicobacter pylori* (HP), with wide geographical variation in prevalence [6]. HP is associated with socioeconomic factors including low family income, living in rural areas, crowded housing, and contaminated drinking water [7]. Noninvasive laboratory tests used for HP include serology, urea breath, and stool antigen tests [8]. Large meta-analyses have converged on a significant association between HP infection and HG (pooled ORs 1.3–3.3) [9, 10]. The majority of HP and HG association studies have been performed in regions with a high prevalence of infection, most commonly case-control studies with a median number of ~100 subjects [10, 11]. There has been substantial variation in the detection of a significant association and its magnitude, in part related to different approaches to the diagnosis of HG, selection of HP testing modalities, and likely other environmental factors [10, 11]. Positive serology testing can reflect past exposure as well as current infection; the former is a higher likelihood in populations with endemic HP infection. Studies of HP and HG based in the United States are comparatively few. Two studies of separate Hispanic populations in California did not reveal a significant association of HP and HG [12, 13]. A Wisconsin-based study also found no significant association of HP and HG, although race was identified as a significant factor [14].

*H. pylori* infection has been associated with pregnancy-related disorders including iron deficiency anemia, thrombocytopenia, fetal malformations, miscarriage, preeclampsia and fetal growth restriction [15]. Data regarding the relationship of HP infection to metabolic disturbances in nausea and vomiting of pregnancy are sparse [16]. HP has been associated with hyperemesis and more severe patterns of vomiting [17]. Therefore, one may predict effects of HP on serum electrolytes such as more marked alkalosis, hypochloremia, and hypokalemia. The impact of dual HP positivity and NVP on maternal health and neonatal outcomes has been investigated in fewer studies. In a Netherlands cohort, pregnant subjects with positive HP serology were more likely to report daily vomiting and exhibit lower total weight gain [17].

Outstanding questions are whether the association of HP exposure and NVP extends to US populations and how their combination impacts pregnancy outcomes. We designed a study of *H. pylori* test results, diagnoses of nausea and vomiting or pregnancy, and pregnancy outcomes at a major US academic medical center and validated the findings on a large multi-institutional database. Our goals were to examine the association of past or current *H. pylori* infection with nausea and vomiting of pregnancy (including HG) in a diverse U.S. population. We hypothesized that a history of a positive *H. pylori* test is a risk factor for more severe nausea and vomiting of pregnancy, with laboratory evidence of metabolic disturbance and adverse pregnancy outcomes.

## 2. Methods

### 2.1. Study Design and Data Collection

A human subject study protocol was approved by the University of Washington Institutional Review Board (STUDY00012501), and a validation protocol was approved by the University of Iowa IRB (202108127). We designed a retrospective study of pregnant subjects who had health care at the University of Washington Medical Center or Harborview Medical Center over 10 years (2010–2020) and validated the findings on a large multi-institutional database. Strengths of the discovery cohort include access to individual subject-level data with uniform laboratory and outcome metrics, and minimal missing data. Relatively low numbers of subjects with HP testing in the discovery cohort (*n* = 332) were a limitation for statistical power in detecting small effect sizes. The use of the independent, larger multi-institutional database validation cohort increased statistical power and allowed testing for consistent associations that are generalizable to the U.S. population. Limitations of the validation cohort were access to aggregated and deidentified data and likely nonuniformity across the participating institutions in clinical practices such as diagnosis of NVP and HP testing patterns.

The University of Washington affiliated hospitals discovery cohort subjects had an ICD code indicating pregnancy associated with an encounter, as identified in an enterprise data warehouse (EDW). We obtained demographic data and ICD codes corresponding to hyperemesis gravidarum and vomiting in the EDW. Nausea and vomiting in pregnancy were defined as ICD code indicating vomiting in pregnancy (ICD-10 O21^∗^) or vomiting (ICD-10 R11^∗^) within the gestational period. Hyperemesis gravidarum was defined by an ICD code indicating hyperemesis gravidarum with metabolic disturbance (ICD-10 O21.1). Infant birth weights and gestational age at birth were obtained from the EDW where available, and missing data were filled in by manual chart review (Epic, MINDscape). Race and ethnicity were based on patient responses recorded in the EHS which allows multiple race selections per patient. The laboratory information system (Sunquest) was used to obtain all serum electrolyte results from the date of pregnancy ICD code assignment to 300 days after. This time range was selected to assess serum electrolyte changes during pregnancy. HP results were obtained from 5 years before the timestamp for the ICD code indicating pregnancy, and up to 300 days after. HP tests included culture, urea breath test, stool antigen, and serology (IgG) [8]. We selected this time frame for HP results to ensure sufficient case numbers to assess associations with NVP and preterm delivery. Importantly, positive HP in this context reflects exposure to *Helicobacter pylori* before or during pregnancy and is not necessarily active infection at the time of pregnancy. Distinguishing active and past infection in retrospective database studies is problematic for several reasons, including difficulties with an accurate assessment of outpatient antibiotic administration, confirmation of completed antibiotic courses, confirmation of successful eradication, and exclusion of reinfection. Since current guidelines recommend eradication therapy for all infected patients in the general population [18], untreated active infections (positive HP) during pregnancy in these cohorts are expected to be uncommon.

The validation cohort was derived from a multi-institutional database (TriNetX, https://trinetx.com/) with aggregated and deidentified data. Vomiting in pregnancy and hyperemesis gravidarum are defined by ICD codes O21^∗^ and O21.1, respectively. Only HP stool antigen and urea breath test results from 5 years prior to and 300 days after the timestamp for the ICD code indicating pregnancy were included. Serology results were excluded due to a lack of uniform titer thresholds for interpretation. Aggregated serum electrolyte laboratory data from the date of the ICD code indicating pregnancy to 300 days after were included. Aggregated race and ethnicity were taken as entered in the Trinetix database. The likelihood of differing race and ethnicity assignment procedures at the various participating institutions is high. Fetal and neonatal outcomes were assessed using ICD codes only: low birth weight (ICD P07^∗^), preterm delivery (ICD O60.1), and second trimester preterm delivery (ICD-O60.12). Potential confounding factors were incorporated into the multivariate logistic regression model using ICD codes for obesity/overweight diagnosis (ICD E66^∗^), multiparity-associated diagnoses (ICDs Z64.1, O09.4, O09.52, and O09.62), housing and economic problems (ICD Z59^∗^), education and literacy problems (ICD Z55^∗^), high-risk pregnancy (ICD O09^∗^), and tobacco, alcohol, or other drug use (ICDs Z72.0, F10-19). These factors are subject to coding biases and are imperfect markers of socioeconomic status and substance use. ICD code definitions and prior literature supporting associations with HP, NVP, and/or preterm delivery are listed in Table 1.

### 2.2. Data Analysis and Statistics

**Figure 1:**
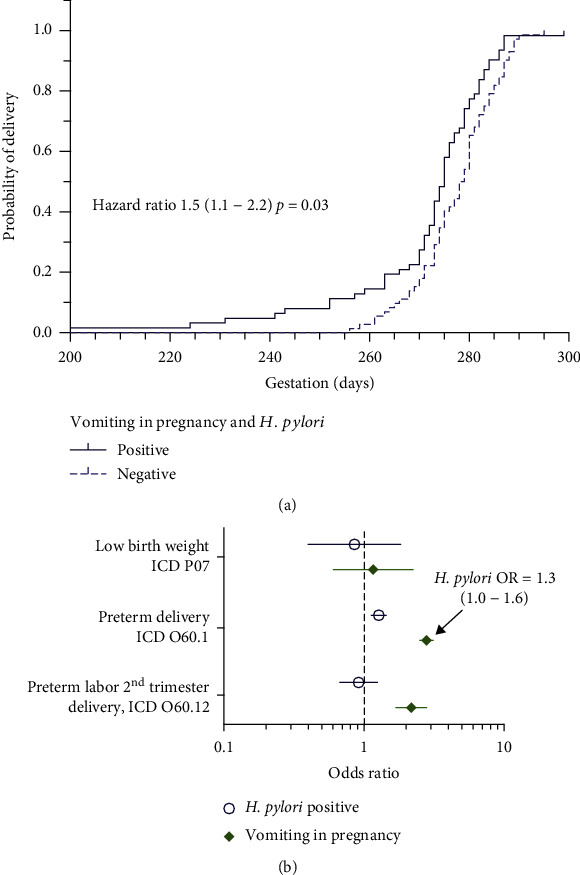
Vomiting in pregnancy and positive *H. pylori* testing results interact to predict risk of preterm delivery. (a) Among subjects in the University of Washington discovery cohort with vomiting in pregnancy, positive HP test result was associated with delivery at earlier gestational age (median 275 days versus 279 days). Hazard ratio and *p* value represent the Mantel-Cox log rank test. (b) Pregnancy outcome ICD-10 codes were correlated to *H. pylori* test results (up to 5 years before pregnancy) and vomiting in pregnancy. Error bars represent 95% confidence intervals of the odds ratio, and absence of overlap with odds ratio 1 (dotted line) is considered statistically significant. Vomiting in pregnancy is associated with preterm labor, including 2^nd^ trimester delivery. Positive HP tests are associated with preterm labor, both in the pregnant population as a whole and within the subset having vomiting in pregnancy (inset).

Primary data assembly and analysis were performed with RStudio (http://www.rstudio.com/). Laboratory data were plotted with mean and standard deviation using Prism 9 (GraphPad, La Jolla, CA). Odds ratios were calculated with confidence intervals as previously described [19]. Pairwise comparisons were performed using the student's *t*-test. Kaplan-Meier plots were assessed with the Mantel-Haenszel hazard ratio calculation and statistical significance testing with Mantel-Cox test. The proportional hazards assumption was tested with a test for independence between scale Schoenfeld residuals and time using R and package “survival.” A nonsignificant *p* value of 0.3 corresponding to the model in Figure 1(a) supports the proportional hazard assumption. Multivariate logistic regression was performed in RStudio, using the “glm” function with family parameter “binomial”. Logistic regression was selected to accommodate mixed categorical and continuous dependent and independent variables and to generate adjusted odds ratios readily compared to the prior literature. In all multivariate analyses, only subjects with complete data were included, reflected as *n* values in the table. For example, the 11,546 subjects lacking an HP result were excluded from analyses incorporating this variable. Odds ratio estimates for the interaction of HP and NVP were calculated with the interaction R package [20]. Race and ethnicity and insurance type distributions were compared using Chi-square tests. Association of hyperemesis gravidarum ICD codes and *H. pylori* testing results was tested with the Fisher exact test and by examination of odds ratio confidence intervals that did not include 1. Statistical significance was defined as *p* < 0.05.

## 3. Results

The discovery cohort consisted of 11,878 subjects with an ICD code indicating pregnancy who received medical care at the University of Washington affiliated hospitals in the 10-year interval 2010-2020 (Table 2). Among these, 2750 (23%) had an ICD code indicating vomiting in pregnancy, 1048 (9%) had an ICD code indicating hyperemesis gravidarum, 332 (3%) had *Helicobacter pylori* testing in the 5 years prior to or during pregnancy, and 299 (3%) had serum electrolyte testing during pregnancy. Subject self-reported race and ethnicity indicators showed significant association with both hyperemesis gravidarum and *H. pylori* testing results. For instance, subjects reporting Black race (including combinations with other categories) had vomiting in pregnancy rates >30% and a 62% HP positivity rate in this cohort (Table 2). The health insurance type documented also correlated with varying rates of vomiting in pregnancy and *H. pylori* test positivity; highest rates of both correspond to Medicaid coverage (Table 2).

**Figure 2:**
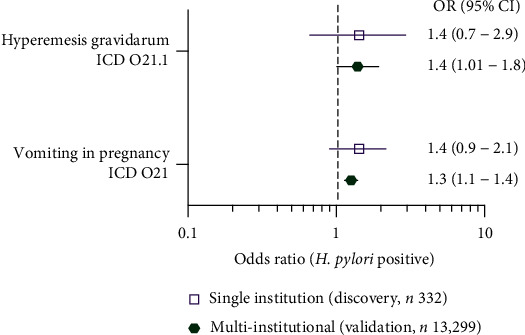
Positive *H. pylori* test results are associated with risk of vomiting in pregnancy and hyperemesis gravidarum in US populations. A positive *H. pylori* test result within 5 years preceding pregnancy is more frequently seen in patients with hyperemesis gravidarum or vomiting in pregnancy (odds ratio (OR)1.3-1.4). *n* values represent the number of subjects with pregnancy and *H. pylori* test results in the University of Washington discovery and large multi-institutional validation cohorts. Results are considered significant if the 95% confidence interval does not include 1 (no risk).

We first examined the evidence for an association between positive HP and NVP or HG. No significant association was detected among the 332 subjects who had HP testing in our discovery cohort (Figure 2(a), Table S1). However, the statistical power is limited by the number of subjects with an HP test result (45%), and there was a trend toward a small magnitude positive correlation (OR 1.4) as seen in prior meta-analyses [10]. An identical association analysis in the larger multi-institutional cohort (13,299 subjects with pregnancy in a 10-year interval 2010-2020, and an HP test up to 5 years before or during pregnancy) showed a similar trend with statistically significant associations of positive HP result to vomiting in pregnancy (OR 1.3, 95% CI 1.1-1.4) and HG (OR 1.4, 1.01-1.9) (Figure 2(a)). We conclude that there is a small magnitude significant association of positive HP and vomiting in pregnancy, including HG in this US population.

**Figure 3:**
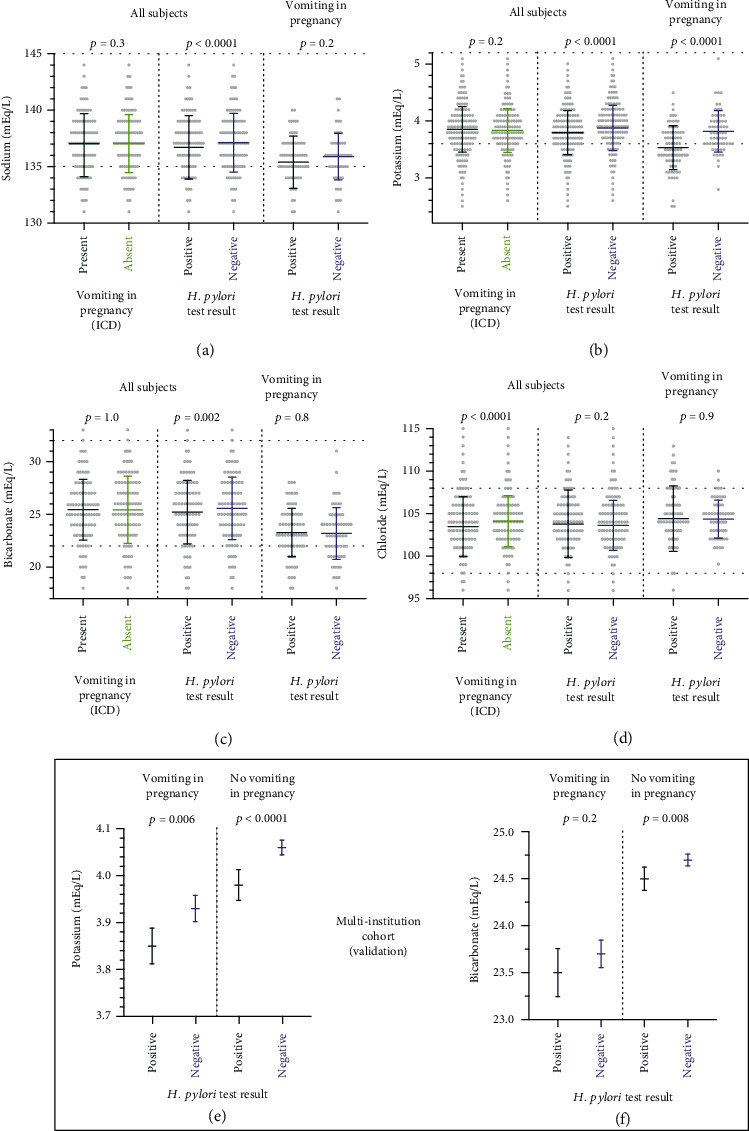
Low serum potassium and other electrolyte alterations are related to *H. pylori* test results and vomiting in pregnancy. (a–d) Serum electrolyte data from the University of Washington discovery cohort are stratified by diagnosis of vomiting in pregnancy and *H. pylori* test result (up to 5 years before pregnancy). Sodium and potassium distributions for all patients were in the lower end of the reference range, likely reflecting hemodilution during pregnancy. Subjects with vomiting in pregnancy had lower serum chloride, and positive HP test correlated with lower sodium, potassium, and bicarbonate. Among patients with vomiting in pregnancy, cooccurrence of a positive HP test associated with low serum potassium. (e, f) The multi-institutional validation cohort confirmed associations of low serum potassium with positive *H. pylori* test, lowest in patients with vomiting in pregnancy. Serum bicarbonate was modestly lower in patients with vomiting in pregnancy, and HP test result was significantly correlated to bicarbonate only in subjects without vomiting. Horizontal lines with error bars represent mean and standard deviation. Points represent individual values, which were not accessible in the multi-institutional cohort (aggregate deidentified data only). Statistical testing was the Student's *t* test.

Serum electrolyte test results were examined to evaluate the potential relationships of NVP and HP to metabolic disturbances (Figure 3). Three comparisons are depicted for each electrolyte, designed to detect correlations with NVP, HP, and HP within the group of patients with NVP (i.e., interaction of HP and NVP). Overall, the mean serum potassium and bicarbonate levels trended toward the low end of the reference interval (Figures 3(b) and 3(c)). This pattern has been observed in normal pregnancy and is likely related to the physiologic expansion of fluid volumes [21]. Positive HP in the discovery cohort, regardless of ICD codes indicating vomiting, was correlated with lower-serum potassium (mean difference 0.1 mEq/L, *p* < 0.0001) and lower bicarbonate (mean difference 0.25 mEq/L, *p* = 0.008). Among patients with an ICD code indicating NVP, positive HP was associated with lower serum potassium (mean difference 0.08 mEq/L, *p* = 0.006) (Figure 3(b)). Although the magnitude of the mean serum potassium difference is small, it corresponds to more frequent hypokalemia (53% among HP-positive subjects and 19% among HP-negative subjects with NVP), defined as a value below the reference interval, which may prompt clinical intervention. Similar patterns of serum potassium and bicarbonate laboratory values were measured in the multi-institutional cohort (Figures 3(e) and 3(f)), validating the finding of altered serum electrolytes in patients with positive HP and NVP.

We next examined birth outcomes in the discovery cohort. We focused on outcomes of preterm delivery and infant birth weight for gestational age in the discovery cohort because they are numeric values accurately documented in the EHR for all subjects, independent of ICD coding. Outcomes of small for gestation age (weight for age z − score < −1.28) and preterm delivery were modeled in relation to HP test result, NVP, or the combination thereof, as well as potential confounding factors of race and insurance type (Table 3). The potential confounding variables included in the model are not exhaustive but selected to account for their observed associations with NVP and HP in this discovery cohort (Table 2). HP positivity in the setting of NVP emerged as a significant risk factor (adjusted OR 8.8 [1.0-76], *p* = 0.03) for preterm delivery in the discovery cohort. Corresponding to this finding, a Kaplan-Meier plot (Figure 1(a)) illustrates that a positive HP correlates with delivery at a modestly lower gestation age among patients with vomiting in pregnancy (HR 1.5, *p* = 0.03).

The risk for corresponding outcomes of low birth weight and preterm delivery was assessed in the validation cohort with odds ratio calculations and 95% confidence intervals (Figure 1(b)). Both HP positivity (OR 1.3, 1.1-1.4) and NVP (OR 2.8, 2.5-3.1) were associated with risk of preterm delivery, and the latter for delivery in the 2nd trimester (OR 2.2, 1.7-2.8). To assess the effects of combined HP positivity and NVP on preterm delivery as observed in the discovery cohort (Table 3), we also calculated an odds ratio for HP among the subset of subjects positive for NVP. Among patients with NVP, positive HP was associated with an additional increased risk of preterm delivery (OR 1.3, 1.0-1.6; Figure 1(b), inset). We constructed a multivariate logistic regression with the larger validation dataset which also showed increased preterm delivery risk (adjusted OR 1.4 [1.0-1.9], *p* = 0.03) among subjects with both NVP and positive HP (Table 3). Potential confounding factors of multiparity, socioeconomic status, substance use, and high-risk pregnancy (Table 1) were included in the model. Potential confounding variables were chosen based on their prevalence, known associations with HP and/or NVP, and ready accessibility in the aggregated validation cohort database. As expected, multiparity-related diagnostic codes independently correlated with an increased risk of preterm delivery (adjusted OR 1.6 [1.3-1.8]). As crude markers of socioeconomic status, diagnostic codes for housing, economic, literacy, or education problems were also associated with a higher risk of preterm delivery (adjusted OR 3.1 [2.3-4.1]).

We conclude that both NVP and positive HP within 5 years are associated with delivery at lower gestational age (preterm delivery) in US populations. Furthermore, the combination of positive HP and NVP increases the risk of preterm delivery and prematurity. The interaction of NVP and positive HP in predicting preterm delivery was not readily explained by markers of obesity, socioeconomic status, multiparity, or substance use.

## 4. Discussion

The association of *Helicobacter pylori* exposure and NVP (including HG) extends to a large US population, a finding not previously reported in the literature to our knowledge. The effect size is small (OR 1.3-1.4, Figure 2(a)), consistent with a recent meta-analysis of 38 worldwide studies (OR 1.348) [10], which explains why the association trend is only detectable with statistical confidence in a large multi-institutional cohort. Lower serum potassium emerged in pregnant subjects with exposure to *H. pylori*, more pronounced in those with NVP. These findings indicate that past exposure to *H. pylori* can amplify metabolic disturbances in the setting of NVP. While the magnitude of mean serum potassium difference related to HP exposure is small (~1 mEq/L), it does result in substantially more diagnoses of hypokalemia. Pregnancy-related steroid hormones have been implicated in NVP and electrolyte shifts in pregnancy, and others have hypothesized that HP may influence both through hormonal mechanisms [15, 22]. However, this hypothesis remains untested. Importantly, we detected impact of the combination of NVP and *H. pylori* exposure on birth outcomes, particularly preterm and second trimester delivery.

We observed substantial variability in the prevalence of NVP and *H. pylori* exposure in subpopulations stratified by self-declared race and health insurance type (Table 2). This pattern is compatible with variable detection of significant associations of HP and HG in prior studies among distinct populations [9–11, 23]. Others have observed that the strength of association also varies by geographic location [9]. In our opinion, this suggests that *H. pylori* infection may not be directly causative of HG in most cases, and other factors influence risk. We selected candidate confounding factors with high prevalence and published prior evidence for associations with NVP, HP, and/or preterm delivery (Table 1). Importantly, the combination of HP exposure and NVP diagnosis still significantly contributed to preterm delivery risk after adjustment for race and ethnicity, insurance type, obesity/overweight, multiparity, socioeconomic status markers, substance use, and high-risk pregnancy (Table 3). As in all retrospective cohort studies, unmeasured confounding remains a possible explanation for the associations between NVP, HP, and preterm delivery. HP and some pregnancy outcomes associated with HG including lower birth weight and gestational age are linked with low socioeconomic status [24–26]. However, HG itself appears to be more prevalent in patients of higher socioeconomic status [3]. Associations of HP exposure, NVP and their combination with preterm delivery persisted after adjustment for crude covariates of socioeconomic status: insurance type and ICD codes for education, literacy, housing, or economic problems (Table 3). Thus, confounding markers of socioeconomic status do not provide a simple explanation of HP and HG association in our cohorts.

Hypokalemia is a feature of metabolic disturbance in hyperemesis gravidarum [27]. A possible explanation for the observed association with HP could be more severe HG-related symptoms, i.e., increased vomiting and metabolic alkalosis. However, we observed significantly lower serum potassium values in all pregnant subjects with a positive HP test, including those without a diagnosis of NVP (Figure 3). HP exposure alone is associated with lower average serum potassium in pregnancy, and this effect is amplified by concurrent vomiting in pregnancy.

A previous very large cohort study (8 million pregnancies) detected a small increased risk of preterm delivery (OR 1.1) and low birth weight (OR 1.1) in pregnant subjects with HG [3]. We made a compatible observation of the increased risk of preterm labor and delivery in subjects with NVP, although the magnitude of risk in our study (OR 2.8) was substantially higher. We add that in the context of NVP, exposure to *H. pylori* compounds the risk of preterm delivery and prematurity (Table 3 and Figure 1). A possible explanation for this interaction is that prior *H. pylori* infection promotes severe vomiting in pregnancy, greater metabolic disturbance, and increased risk of preterm delivery.

Implications of this study are that patients with prior exposure to HP are at increased risk of NVP and HG, metabolic disturbance, and preterm delivery. Assessing HP status likely has prognostic value for outcomes of NVP and preterm delivery. The association between a positive *H. pylori* test and a higher incidence of hyperemesis gravidarum is not sufficient to imply that eradication therapy should be implemented as part of prenatal care, particularly given the relative contraindications of some components of standard therapy in pregnant patients [28]. Evidence for symptomatic improvement of hyperemesis with eradication during pregnancy is limited to case series [29, 30], and to our knowledge, a beneficial effect in terms of pregnancy and birth outcomes has not been shown. While chronic *H. pylori* infection is associated with numerous adverse health outcomes and should be diagnosed and eradicated in the general population [18, 31], compelling evidence (e.g., prospective trials) for doing so during pregnancy is currently lacking.

Further study is needed to determine whether *Helicobacter pylori* is causally linked to NVP and the underlying mechanisms for this phenomenon. Benefits of treatment for active HP during pregnancy, particularly in the setting of NVP, remain to be demonstrated in controlled prospective studies.

### 4.1. Strengths and Limitations

Strengths of the study include a large study population and validation of all major single-institution findings in an independent cohort. These features add confidence to measurements and imply generalizability to a larger US population. This study has several limitations. We emphasize that like all retrospective cohort studies, the relationships of HP, NVP, and preterm delivery detected in this study are associative and not necessarily causal. While the retrospective design allowed study of a large cohort over 10 years, the analysis is partially dependent upon accurate diagnostic coding (ICD). This was mitigated in the discovery cohort by focusing on pregnancy outcomes (gestational age at delivery and infant birth weight) that are independent of diagnostic coding. Eradication therapy for HP was not assessed due to unreliability of available medication data. Since *H. pylori* and serum electrolyte testing were performed in a minority of subjects; there are likely biases in the patients selected for testing, such as confounding disease processes prompting clinical evaluation for electrolyte disturbances. However, similar biases of selection for testing are expected among the groups compared in this study. For example, all subjects in the multivariate analysis were clinically selected to undergo HP testing and are only compared based on the test result. The statistical power of multivariate regression and HP and HG interaction analyses was limited by the number of subjects with complete data.

## Figures and Tables

**Table 1:** Diagnostic codes and prior evidence for associations with nausea and vomiting of pregnancy, *H. pylori*, and preterm delivery. The cited literature in this table represents manual selection and is not intended to reflect a complete or systematic literature review. The associations may be either positive or negative and were not necessarily causative or detected in all relevant prior studies. Asterisk indicates that all subclassifications under the indicated category were included.

Diagnosis	ICD codes	Association evidence in prior studies		
		NVP (Ref.)	HP (Ref.)	Preterm delivery (Ref.)
Vomiting in pregnancy	O21^∗^ R11^∗^	N/A	Yes [9, 10]	Yes [2]
Hyperemesis gravidarum	O21.1			
Low birth weight	P07^∗^	Yes [17]	Yes [15]	Yes [32]
Preterm delivery	O60.1	Yes [2]	Yes [33]	N/A
Second trimester preterm delivery	O60.12			
Obesity/overweight	E66^∗^	Yes [34]	Yes [35]	Yes [36]
Multiparity-associated	Z64.1 O09.4	Yes [37]	No	Yes [38]
Housing and economic problems	Z59^∗^	Yes [3]	Yes [24]	Yes [26]
Education and literacy problems	Z55^∗^			
Tobacco, alcohol, other drug use	Z72.0 F10-19	Yes [34]	Yes [39]	Yes [26]
High-risk pregnancy	O09^∗^	Yes [40]	No	Yes [41]

**Table 2:** Vomiting in pregnancy and *H. pylori* test results vary by race/ethnicity and type of health insurance. Subject numbers are shown for the University of Washington Medicine Discovery cohort. The presence of an ICD code indicating vomiting in pregnancy and result of *H. pylori* testing within 5 years of pregnancy each varied by race/ethnicity and health insurance type. Race and ethnicity are as reported by the patient, and one patient may be counted in multiple categories. Age differences are tested with the Kruskal-Wallis test, and race/ethnicity and health insurance differences are tested with a Chi-square test.

	Vomiting in pregnancy			*H. pylori* test result			
	Yes	No	*p*	Positive	Negative	None	*p*
Subjects, *n*	2750	9128	NA	151	181	11546	NA
Age *median years (quartiles)*	32 (27–36)	33 (29–37)	0.5	32 (28–37)	34 (29–37)	32 (28–36)	0.01
Race and ethnicity^†^(self-declared), *n*			<0.001				<0.001
American Indian/Alaska Native	26	104		1	0	129	
Asian	458	1855		28	31	2254	
Black	560	1040		46	28	1526	
Latino	253	721		6	11	957	
Native Hawaiian/Pacific Islander	41	103		1	1	142	
White	1513	5417		31	77	6876	
Unknown	106	495		6	4	591	
Health insurance type, *n*			<0.001				<0.001
Commercial	691	2412		21	32	3050	
Medicaid	529	1115		41	20	1583	
Medicare	9	22		0	1	30	
Other/unknown	1482	5538		51	85	6883	

^†^Subjects declaring multiple races or ethnicities are counted in multiple categories.

**Table 3:** *H. pylori* status is related to pregnancy outcomes in patients with vomiting in pregnancy. Pregnancy outcomes were correlated to vomiting in pregnancy-related diagnoses, laboratory test results, and demographic features identified in this study. Interaction analysis between diagnosis of vomiting in pregnancy and *H. pylori* test result within 5 years of pregnancy was performed using multivariate logistic regression models. Interaction of NVP diagnosis and positive HP result correlated to increased risk of preterm delivery in both discovery and validation cohorts. See Figure 1 for a graphical representation of gestational age at delivery. Blue text indicates lower-order effects of NVP or HP in isolation. Interpretation of these is complex due to inclusion of the NVP^∗^HP interaction term. An adjusted odds ratio confident interval not including 1 and p values less than 0.05 were considered significant.

	Small for gestational age (z-score< -1.28)		Preterm delivery (<37 weeks)	
	Adjusted odds ratio [95% CI]	*p*	Adjusted odds ratio [95% CI]	*p*
Discovery cohort *n 297*				
Vomiting in pregnancy^†^	1.1 [0.3–3.1]	0.9	0.1 [0.0–0.6]	0.04
*H. pylori* positive^†^	0.6 [0.1–1.8]	0.4	0.8 [0.3–1.8]	0.5
NVP^∗^HP positive	1.7 [0.3–10.4]	0.6	8.8 [1.0–76]^‡^	0.03
Race and ethnicity	0.9 [0.3–2.2]	0.8	1.0 [0.4–2.2]	1.0
Medicaid insurance	0.8 [0.3–2.2]	0.7	1.2 [0.5–2.9]	0.6
Validation cohort (*n* = 11,995)				
Vomiting in pregnancy^†^	n.d.		2.4 [1.3-4.3]	0.006
*H. pylori* positive^†^	n.d.		0.6 [0.3–0.9]	0.03
NVP^∗^HP positive^‡^	n.d.		2.3 [1.3–4.3]	0.02
Obesity/overweight	n.d.		1.6 [1.3–2.1]	<0.001
Multiparity-associated diagnosis	n.d.		3.5 [2.1–5.5]	<0.001
Education, literacy, housing or economic problem	n.d.		3.1 [2.3–4.1]	<0.001
Substance use diagnosis	n.d.		1.1 [0.8–1.4]	0.6
High-risk pregnancy	n.d.		1.1 [0.9–1.3]	0.6

^†^Interpretation of lower order effects, NVP or HP in isolation, is complex due to inclusion of the NVP^∗^HP interaction term. ^‡^Odds ratios reflect the effect of positive HP within the strata of subjects with NVP.

## Data Availability

Data will be made available upon reasonable request to the corresponding author. The TriNetX database can be accessed at https://trinetx.com/.

## Supplementary Materials

Supplementary Materials Table S1: two by two contingency tables associating *H. pylori* exposure with nausea and vomiting of pregnancy and hyperemesis gravidarum.
