# Supplementary material for: Helicobacter pylori Exposure in Nausea and Vomiting of Pregnancy Increases Risk of Preterm Delivery
Source: Infect Dis Obstet Gynecol. 2023 Sep 28;2023:6612268. doi: 10.1155/2023/6612268 (PMC10555503; doi:10.1155/2023/6612268)
Supplement: Supplementary Materials — Table S1: two by two contingency tables associating H. pylori exposure with nausea and vomiting of pregnancy and hyperemesis gravidarum. [file 6612268.f1.pdf]

Discovery cohort			
	***H. pylori* result**		
	**Positive**	**Negative**	**Chi square p**
Hyperemesis gravidarum	17	14	0.3
No hyperemesis gravidarum	134	167	
Nausea and vomiting of pregnancy	64	78	0.9
No nausea and vomiting of pregnancy	87	103	
**Validation cohort**			
	***H. pylori* result**		
	**Positive**	**Negative**	**Chi square p**
Hyperemesis gravidarum	49	151	0.04
No hyperemesis gravidarum	2471	10628	
Nausea and vomiting of pregnancy	646	2314	<0.001
No nausea and vomiting of pregnancy	1874	8465	

Table S1. *Two by two contingency tables associating H. pylori exposure with nausea and vomiting of pregnancy and hyperemesis gravidarum.*
